# International humanitarian law violations in northern Uganda: victims' health, policy, and programming implications

**DOI:** 10.1057/s41271-023-00407-8

**Published:** 2023-04-20

**Authors:** Anastasia Marshak, Teddy Atim, Dyan Mazurana

**Affiliations:** 1 Feinstein International Center, Tufts University, Boston, MA USA; 2 York University, Toronto, ON Canada; 3 Feinstein International Center, Tufts University, 75 Kneeland St, 8th Floor, Boston, MA USA

**Keywords:** Serious violations of International Humanitarian Law, Uganda, Health access, Health policy, International Criminal Court

## Abstract

Experience of serious violations of International Humanitarian Law (IHL) results in complex physical disability and psychosocial trauma amplifying poverty and multi-generational trauma and impeding long-term recovery. We use data from a representative sample of victims in the case *Prosecutor V. Dominic Ongwen* brought before the International Criminal Court. Thirteen years after the 2004 massacre, the victims were significantly worse off than the general war-affected population that did not experience serious violations of IHL. The differences in health and wellbeing persisted for individuals and their households, including children born after the massacre. The victims have significantly lower availability of appropriate health services and medications, including significantly greater distance to travel to these services. These findings call attention to the needs of people having experienced IHL violations, for provision of physical and emotional trauma care to allow for recovery, and better understanding of the short- and long-term impacts of IHL violations.

**Supplementary Information:**

The online version contains supplementary material available at 10.1057/s41271-023-00407-8.

## Key messages

- Conflict has long lasting and profound impacts. Thirteen years after a massacre, individuals and members of their households who survived experienced far more physical disability, direct negative effects on their livelihoods, reduced financial resources, and increased food insecurity–compared to the general war affected population that did not experience a human rights violation.
- The effect of surviving a massacre has a multi-generational association with worse outcomes. Children not yet alive during the massacre but born into victim households have lower school enrollment and attendance rates than their peers.
- Despite experiencing extremely high levels of disability and poor psychosocial wellbeing, the victim population lacks appropriate physical and emotional trauma care and therapeutic treatment, inhibiting recovery and leading to multi-generational differences from the general population.
- Better recognition of human rights violations and the impact of the experience on wellbeing can improve provision of appropriate health services, therapeutic treatment, and trauma care.

## Introduction

Humanitarian crises—an event or a series of events that are threatening in terms of health, safety or well-being of a community or large groups of people—are often caused by complex and protracted conflicts, meaning conflicts that endure over several years. Conflicts, as opposed to other humanitarian crises, drive 80% of the cost of all material and logistic assistance needed during and after a humanitarian crisis [1]. Current examples include the Ukraine, Democratic Republic of Congo, Afghanistan, Yemen, Sudan, South Sudan, Syria, and Somalia. Conflicts contribute to the global prevalence of people living with disability [2] and psychosocial trauma.

Conflicts can also lead to experiences of serious violations under International Humanitarian Law (IHL) (also known as war crimes). IHL is a set of rules which seek to limit the effects of armed conflict, specifically protecting persons who are not or are no longer participating in the hostilities and restricts the means and methods of warfare. Serious violations of IHL can take place in international or non-international armed conflicts. Violations of IHL are considered serious if they endanger protected persons (such as civilians) or objects (such as infrastructure) or breach important values. Specific war crimes or serious violations of IHL include murder, attempted murder, torture, enslavement, outrages upon personal dignity, pillaging, destruction of property and persecution, forced marriage, rape, sexual slavery, enslavement, forced pregnancy, and conscripting children under the age of 15 [3].

Having experienced serios violations of IHL inhibits long term recovery. The most frequently quoted figure, although not updated since 2004, is that 15% of the world population experiences a disability [4]. More recent evidence points to an increase in that proportion, driven by an aging population, climate disasters, and conflict [2]. A 2018 Needs Assessment from Syria following the war shows that 99% of all individuals above 59 years of age had a disability, as did almost one-fifth of all children under the age of 18 [5]. To improve policy and programming in conflict affected contexts, we need to better understand the impact of serious violations of IHL and resulting experience of physical and mental disability and overall wellbeing and to investigate whether appropriate health services are available to contribute to recovery and prevent multi-generational trauma.

Research on the impact of war violence on civilian populations has focused primarily on mortality, malnutrition, and infectious disease and shows worse outcomes for war-affected populations [6–9]. A body of literature is growing on the role of specific forms of serious violations of IHL on mental health outcomes for victims and survivors [10–13], with less attention to the physical consequences and impacts on livelihoods [14–17]. Studies found that victims of violations or abuses, who vary by sex and age, often have diminished mental and physical health and quality of life, reduced economic and educational opportunities, and experience stigma by their families or communities. Some studies also found that female victims, in particular, may have experienced increased domestic and sexual violence post conflict [18–20].

There is less focus in the literature on the availability and use of appropriate health care for victims of war violence. Most research focuses on combatants showing that in low resource countries, there is poor availability of therapeutic health care [18, 21, 22]. Even in high resource countries like the United States, veterans from wars in Iraq and Afghanistan seeking health care are so abundant that they have overwhelmed the Veterans Health System with their serious health care needs, resulting in inadequate overall care [23, 24]. Most health-focused studies on war violence against civilians focus on framing, diagnosing, and describing the mental and psychosocial health of their study populations [10, 12, 25–27].

To improve our understanding of the association of serious violations of IHL with outcomes of well-being, we draw on a novel set of data originally collected by the authors as part the case *Prosecutor V. Dominic Ongwen* in conflict-affected Uganda brought before the International Criminal Court (ICC). The ICC investigates and, where warranted, tries individuals charged with serious violations of IHL. The ICC is governed by an international treaty called the Rome Statute, which establishes the ICC as a permanent institution that has the power to exercise its jurisdiction over persons for the most serious crimes of international concern. It serves as a complementary institution to national criminal jurisdictions. The ICC put Dominic Ongwen on trial in 2015 for a series of war crimes committed in northern Uganda (see Supplementary Table S1).

This study documents the physical, material, and psychosocial harm suffered by the victims of Ongwen in massacres committed in three Internally Displaced Camps (IDP), assesses the immediate and repercussive effects on the victims and their households and recommends appropriate responses. We use a unique approach to assess or hypothesize “impact” by comparing data directly from the victims to the general war affected population in the Lango and Acholi sub-region that did not suffer a serious violation of IHL. The use of data from a research study, as opposed to individual witness testimonials, in an international criminal proceeding is novel and offers insights into the long-term (13 years after the event) harms of serious violations of IHL. In the supplementary material, we provide a timeline of events relevant for the massacres in the three IDP camps northern Uganda in 2004 and the methodology used for the data collection and analysis.

## Results

### IHL violations

As a result of the LRA attacks against the three IDP camps in 2004, the 396 living victims experienced an average of 6.9 serious violations of IHL. The experience of IHL indicates that serious violations clustered during the attacks, with half of the victim population (VP) experiencing 6 or more serious violations. Critical for understanding the association of IHL violations with well-being is understanding the collective experience of all members of the household. Ninety-one percent of victims came from a household where a household member, other than the respondent, experienced a serious violation of IHL. On average, each of the VP households experienced 22 serious violations. We present a qualitative narrative of the household level impact described by the VP below (Table 1).

**Table 1:** Direct quotations from victim population

Citation #	Quote	References
1	On the day of the attack at Abok, I had just returned back from hospital because of an earlier LRA attack that had left me wounded. My brother and I had gone to the village [from the camp in Abok] to dig and that is when we were attacked by the rebels. My brother was killed while I was hacked on the head with a machete. On the day of the attack at Odek, my wife and children were set ablaze in the hut, but they managed to escape away. The rebels also looted all our property while destroying some of the things they could not take with them	Male interviewee from Abok
2	Due to the war, our life and the entire community has been destroyed so much. Trauma is still there to date in the families who were affected. Like for us, we lost about 40 people from our close family during the attack. They were all were related to us—from the same lineage. So, life is not the same. There is high level of alcoholism in the community to as a way to help people forget what happened to them. But due to the high level of alcoholism, people are unable to work or function normally. They are not well enough to produce enough and provide for their families, thus poverty is on the increase in most households For me, I had a lot of dreams. I would dream of my sister’s son who I played with together, but he was killed in the attack. Sometimes I would see him in my dream with a panga [machete] coming to kill me so I could go where he is. This is because after his death, I thought about him so much because I felt so lonely and this caused me those dreams	Male interviewee from Lukodi
3	During the time my sister was with the rebels (was abducted the day of the attack and spent seven years in rebel captivity returning with a child), she was wounded on her leg and also had a bullet lodged in her chest. That bullet was removed when she returned home Once the pain starts, she is unable to do even basic things such as farm the land or cook. Because of my sister’s injury and mental condition (spirit attacks), I have to provide for all her needs. That is why we live close to each other so I can support her and her child	Female interviewee from Odek
4	She has to see a doctor all the time but we can’t afford the costs. We also have to check or have her brain examined to ascertain the extent of the damage on her head, but we have not yet done it. We don’t know how…we can’t afford it We have to buy her medication each time she complains of headache, which she has constantly especially when she is exposed in the heat for a long time. If she has taken a long time in the sunshine, that causes her nose to bleed all the more. So, she can’t be exposed in sunshine for long because it will cause her headache and bleeding from her nose. We can’t afford to take her to hospital that can provide her good help	Female interviewee from Abok
5	Because there was no men to help us [after the murder of my father in the Odek attack], we couldn’t produce enough food for the family. We always had food shortages at home. My mother alone couldn’t produce enough on her own to feed the entire family. There was nothing extra to sell to send us to school	Female interviewee from Odek
6	We are unable to continue with school because getting money has become a lot harder today because our main source of livelihoods was lost [in the Lukodi attack]. If my elder brother was here [he was abducted during the attack and never returned], possibly he would be supporting our parents to raise income to send the younger ones to school, as is expected of any elder brother	Male interviewee from Lukodi

### Psychosocial wellbeing

Using the AYPA tool, we observed significant differences between men and women, with women doing worse when it comes to depression, anxiety, and somatic complaints without medical causes. More so, experience of multiple IHL violations had a compounding effect for women (*p* < 0.01), with each additional violation increasing the AYPA by 1 point (out of 96), compared to 0.33 points for men (*p* = 0.09). Of all the IHL violations, the experience of losing a child or having one’s child injured had the greatest contribution to the AYPA (*p* < 0.05), together contributing to 22 percent of the mean score. The association between psychosocial trauma and loss, but particularly the loss of a child comes out clearly in the testimonials (#2 in Table 1). Although we cannot compare the overall AYPA score between the VP and the general population (GP), given that each IHL violation is associated with an increase in the AYPA, we can hypothesize that the VP would have a higher AYPA score, indicating overall worse psychosocial wellbeing.

### Health services availability

Two-thirds of the VP reported having a disability compared to 16% of the GP (Table 2). A VP has on average 2 disabled individuals in the household with 70% of household members classified as dependents, compared to 47% in the GP. Disability can have long lasting effects on livelihood opportunities and income for both the affected individuals and their caretakers (#3 in Table 1).

**Table 2:** Self-reported outcomes by study group and gender of participant (%/mean with confidence interval in brackets)

	Male		Female		Total	
	Victim population	General population	Victim population	General population	Victim population	General population
Have a disability (%)	64.5**	16.6	69.3**	23.6	67.0**	21.1
	[57.7–71.2]	[12.3–20.8]	[62.8–75.9]	[20.0–27.3]	[62.3–71.7]	[18.3–23.9]
Number of people w/disabilities in the household (mean)	2.07**	0.47	2.15**	0.53	2.11**	0.51
	[1.85–2.30]	[0.38–0.56]	[1.92–2.38]	[0.46–0.60]	[1.95–2.27]	[0.46–0.56]
Dependency ratio^a^	0.67**	0.45	0.72**	0.48	0.70**	0.47
	[0.64–0.70]	[0.42–0.48]	[0.69–0.75]	[0.46–0.50]	[0.67–0.72]	[0.45–0.49]
Travel time (minutes)	143**	93	143**	100	143**	98
	[124–163]	[83–102]	[126–161]	[92–109]	[130–156]	[91–104]
‘Access’ for routine health problems (%)	4.6*	9.8	5.5*	11.8	5.0**	11.1
	[1.6–7.5]	[6.4–13.2]	[2.3–8.6]	[9.1–14.6]	[2.9–7.2]	[9.0–13.3]
‘Access’ for serious health problems (%)	3.6	8.3	4.1*	9.2	3.9**	8.6
	[1.0–6.2]	[4.5–12.1]	[1.3–7.0]	[6.7–11.7]	[1.9–5.8]	[6.7–10.5]
Availability (%)	7.4**	19.4	10.9**	20.6	9.7**	20.2
	[4.4–10.4]	[14.9–23.9]	[6.6–15.3]	[17.2–24.1]	[6.7–12.6]	[17.4–22.9]
Financial capacity	4.95**	5.37	4.48*	4.76	4.70**	4.98
	[4.78–5.12]	[5.22–5.52]	[4.31–4.66]	[4.64–4.88]	[4.58–4.83]	[4.88–5.08]
Food insecurity	21.7**	14.0	20.5**	15.4	21.1**	14.9
	[19.8–23.7]	[12.7–15.4]	[18.7–22.4]	[14.4–16.4]	[19.8–22.4]	[14.1–15.7]

***Significant at *p* value < 0.001, **significant at *p* value < 0.01, *significant at *p* value < 0.05

Given the high level of physical harm experienced by the VP population with respect to the GP, we look at ‘access’ to health services for routine and serious health problems. ‘Access’ encompasses availability of appropriate treatment, cost of treatment, and distance needed to travel for treatment all in one question. Compared to the GP, it takes VP households significantly longer (in minutes) to reach a health clinic, VP were significantly less likely to say they can ‘access’ a health clinic that has the services they need for routine and serious health problems, and the VP were significantly less likely to report that the clinic had the necessary services and medication available (Table 2). These differences remain when controlling for other individual and household level characteristics (Table 3).

**Table 3:** Multivariate logistic and linear regression on health access and quality outcomes (*n* = 1210)

	‘Access’ for routine health problems		‘Access’ for serious health problems		Availability		Travel time (minutes)	
	Odds ratio	*p* value	Odds ratio	*p* value	Odds ratio	*p* value	Coef	*p* value
GP (reference: VP)	2.20	0.01	1.99	0.04	2.04	0.00	− 43.16	0.00
Female (reference: male)	1.41	0.16	1.38	0.25	1.16	0.43	− 8.58	0.20
Age	1.00	0.89	1.00	0.86	1.01	0.15	− 0.32	0.10
Educational attainment	1.01	0.68	0.99	0.74	0.99	0.74	− 3.24	0.00
Household size	1.07	0.09	1.06	0.14	0.98	0.49	− 1.92	0.11
Financial capacity	1.08	0.39	1.19	0.10	1.07	0.31	− 4.33	0.08
Food insecurity	0.98	0.08	0.97	0.02	0.99	0.08	0.17	0.49
Constant	0.01	0.00	0.01	0.00	0.04	0.00	252.92	0.00

Using the MSI as indication for the financial capacity to pay for services, we found that the VP population has significantly less assets and hence financial capacity than the GP (Table 2). The more IHL violations experienced by an individual or in a household, the lower their financial resources (*p* = 0.02 and *p* < 0.01, respectively). A mother explains the relationship between IHL violations and financial resources when speaking about her daughter who as a child was severely burned by the LRA during the attack (see #4 in Table 1).

### Long-term effects

More than a decade after the war, the experience of IHL violations is directly associated with food insecurity (Table 2). Citation 5 (Table 1) from a male victim participant shows the link between the attacks and immediate and prolonged food insecurity, including how the breakdown of families exacerbates household food insecurity today.

The association of the serious violations of IHL and worse outcomes is not limited to the VP but affects all members of the household. Boys and girls in VP households are significantly less likely to attend school every day (Table 4). The difference remains for children who were between the ages of 5 and 10 years at the time of the survey, and hence were not alive during the massacre in 2004 (*p* = 0.01) (Citation #6 in Table 1).

**Table 4:** Attendance at schools (means with confidence interval in brackets)

	Victim population	General population
Boys attending school every day (%)	48.6**	65.2
	[42.9–54.4]	[61.0–69.3]
Girls attending school every day (%)	46.4**	62.0
	[40.6–52.2]	[57.8–66.3]

***Significant at *p* value < 0.001, **significant at *p* value < 0.01, *significant at *p* value < 0.05

## Discussion

The findings from the study and how they were used in the case against Ongwen illustrate four key things. First, the extent of serious violations of IHL experienced during the brief massacres in the three IDP camps in 2004 are far greater than what the average household in war affected Lango and Aholi sub-region experienced across the entire 20 + years of war. Second, the possible impacts of IHL violations are compounding and can last for at least a decade after the event, in a multi-generational manner. Third, despite the association of IHL violations and physical disability and psychosocial wellbeing, the affected population has less access to the health services they need, impeding long-term recovery. And fourth, getting data on the association of serious violations of IHL with key outcomes is not just possible but absolutely necessary to represent the experiences of the victims and determine appropriate reparations and national health policy.

Across the 20 + years of war, a representative survey of Acholi and Lango sub-region (using the same questions as our survey), found that on average, individuals experienced 0.34 serious violations of IHL, with those who experienced a serious violation averaging 2.5 violations [20]. In contrast, as a result of the LRA attacks against the three IDP camps in 2004, victims experienced an average of 6.9 serious violations of IHL. To put it more starkly, the VP experienced, on average, 20 times more serious violations of IHL compared to the average war affected person in Acholi and Lango, with children experiencing 14 times more violations than individuals in the sub-region representative survey. On average, each of the VP households experienced 22 serious violations, compared to the average household in Acholi and Lango where members experienced 2.3 violations per household over the 20 years of war [20].

Few studies look at the role and availability of health care for affected populations. Research among IDPs in Gulu and Amuru districts in northern Uganda shows that being ill without medical care had the strongest association with post-traumatic stress disorder and depression, with over half of all respondents having reported these symptoms; less than one-third being able to get the appropriate care [28]. Another study in Gulu district identified a lack of services available to victims of gender-based violence (GBV), specifically limited qualified staff and medical supplies to detect and manage survivors and services offered without ensuring confidential treatment and counseling [29]. A more recent review (2021) found that almost two-thirds of medical care professionals dealing with GBV in Uganda needed additional training and nearly half were uncertain or disagreed that there were clear protocols for care of survivors [30]. A study in three districts in northern Uganda shows how the conflict itself can directly affect the health services required by the affected population with direct attacks on health facilities, looting of medical supplies, and abduction of health providers [31]. While health care access was poor before the war, the limited evidence to date points to an even further weakened health care system that is unable to meet the physical and mental health care needs of the war-affected population. In northern Uganda, Betancourt et al*.* (2009) and Porter (2016) investigated how local government health providers dealt with mental health problems. They have provided important insights into how culture mediates what constitutes ill-health, its sources and manifestations, and solutions people seek to restore their health [32, 33].

The reports on serious violations of IHL shows that individuals and their households affected by a massacre are significantly different from the general war-affected population, in both their experience of IHL violations [20] and the possible impact of those violations. The VP are significantly worse off in terms of their psychosocial and physical well-being; these are directly associated with lower wealth and worse food insecurity, further exacerbated by less access to and lower rates of utilization of health services. The complexity of physical and mental health needs of the war-affected population is rarely addressed and redressed in the northern Uganda context [34]. The data also show that the association of IHL violations with worse outcomes is not only for the individual, but also for those in the households where individuals live, making recovery even more difficult. When it comes to the VP households, the association of IHL violations with worse outcomes is apparent in lower rates of school attendance by children who were not even alive at the time of the attacks. Not only does the continued association with worse outcomes point to possible multi-generational impact, but also exacerbates a possible ‘poverty trap’ (the poor cannot escape their poverty and with lack of resources only get poorer) for affected households created in part by their lower educational attainment [35]. The experiences of serious violations of IHL are compounded, with worse outcomes associated with more IHL violations.

The VP report longer travel time to health facilities, are less likely to have access (measured as a combination of travel, cost, and availability of services) to the services they need for routine or serious health problems and are more likely to report that the health services and medications that they need were not available. The difference in ‘access’ to, availability of, and travel time to appropriate health services between the GP and VP is not likely related to the use of different health centers, given the geographical proximity, but rather an indication of the significantly greater needs of the VP population. The IHL violations experienced by the VP populations is associated with far more complex psychosocial and therapeutic medical needs than services available at already poor and understaffed health centers. An in-depth analysis of the war-wounded within the same population in Uganda shows that there is a lack of the necessary treatments required for their ailments at the health centers, leading affected individuals to become disillusioned and discouraged from seeking the help they need [36]. Previous research in Uganda shows that when health services are targeted, they are much more likely to benefit combatants compared to their non-combatant peers as a result of programming by nongovernmental agencies focus on youth combatants [18].

The study also indicates that data on the association of IHL violations with key outcomes can be collected and serve as a new type of evidence to present before the ICC and to use to improve government policy. Data on the effects of war and serious violation of IHL are critical to give voice to the affected population in international cases and to affect national health policy. Witness or expert testimonies in court cases are traditionally limited to one aspect of the serious violations of IHL that take place. This study provides holistic evidence about the immediate and long-term association between the conflict and worse outcomes. The mix of quantitative data and testimonials allows us to demonstrate the effect of the human rights abuses on the lives of the victims, offer them a means to represent themselves, and offer the court tangible data for determining reparations. Based on the findings, the research team was able to give specific recommendations on how to target services and provide support to the most affected, strengthen psychosocial support and disability support, provide specialized therapeutic health services, provide education support, and the need for physical and monetary compensation for destroyed assets and livelihoods.

The findings have direct implications to international and government actors working in previously conflict affected contexts, not only in Uganda, but across the multitude of protracted humanitarian contexts driven in part by conflict. Once the initial conflict subsides, there is a tendency by development partners to treat populations as `post-conflict’, as though internal differentiation, including varying experiences of serious violations of IHL and related experience of disability and trauma, is unimportant. The findings from this study support the argument that post conflict national health actors and development partners cannot safely assume that everyone is recovering or recovering equally. Instead, these data expose disadvantages previously unappreciated, including persistent health inequality and multi-generational trauma to individuals, households, and communities.

Our study confirms that in post-conflict settings, war continues to be associated with long lasting and profound negative outcomes that need to be directly addressed in rebuilding war-damaged healthcare systems and treating the war-wounded. More attention should be given to the psychosocial and physical health needs of civilians suffering from IHL violations and disability, and the association with their disadvantage in receiving therapeutic treatment over time. Knowledge of the prevalence and negative relationship of war crimes with civilians’ mental and physical health, disability and access to health should be used to help develop more responsive post conflict health and psychosocial policies and services.

### Study and data limitations

Because data collection occurred 13 years after the massacres the sample is only representative of the individuals who survived and did not migrate from the area. Thus, the sample prevalence of serious violations of IHL is likely biased, either downward because individuals who were murdered or disappeared are not included in the sample, or upwards because those who experienced fewer violations might have been able to migrate since the massacre. Because men were overwhelmingly more likely to be affected by direct combat, taken as child soldiers, or disappeared [37], our sample, purposely stratified by sex, is again not representative of the real sex-based experience of IHL violations. Another limitation is that the data is self-reported. Although the findings point to a multi-generational association between IHL violations and worse outcomes, having nutrition and health data collected from the children under five (using standard anthropometrical methods) would have been critical to prove this hypothesis. The analysis also operates under the assumption that prior to the attacks the respondents in the VA survey were more likely to resemble the general population and hence attributes differences in 2018 to the 2004 attack. Without data prior to the attack, we cannot confirm that this is the case; however, given that the GP sample is representative of the area from which the VA population was sampled and that the attacks were not targeted based on household characteristics, we feel confident that we are comparing two populations that more or less resembled each other prior to the massacre.

## Conclusions

The findings from this study were presented by the Legal Representative of the Victims as testimony during Ongwen’s trial to give voice to the victims and for consideration of reparations. More broadly, these findings illustrate how future studies in conflict and post-conflict contexts need to prioritize the collection of IHL violation, disability, and psychosocial trauma data to ensure better programming and policy. Without appropriate services, disability and psychosocial trauma can have long-term consequences beyond the affected individual leading to a growing cycle of poverty.

## Supplementary Information

Below is the link to the electronic supplementary material.

Supplementary file1 (DOCX 240 kb)
