# Supplementary material for: International humanitarian law violations in northern Uganda: victims' health, policy, and programming implications
Source: J Public Health Policy. 2023 Apr 20;44(2):196–210. doi: 10.1057/s41271-023-00407-8 (PMC10232562; doi:10.1057/s41271-023-00407-8)
Supplement: Supplementary file 1 — Supplementary file1 (DOCX 240 kb) [file 41271_2023_407_MOESM1_ESM.docx]

**Journal of Public Health Policy**

Supplementary Materials

**International Humanitarian Law Violations in northern Uganda: victims’ health, policy, and programming implications**

**Running title:** Victims of war in Uganda

Anastasia Marshak*^a*^*, Teddy Atim*^ab^*, and Dyan Mazurana*^a^*

*^a^* Feinstein International Center, Tufts University, Boston, MA, USA

*^b^* York University, Toronto, Ontario, Canada

*^*^*Corresponding Author:

Tufts University, Feinstein International Center, 75 Kneeland St, 8^th^ floor, Boston, MA, USA.

[anastasia.marshak@tufts.edu](mailto:anastasia.marshak@tufts.edu)

**International Humanitarian Law Violations in northern Uganda: victims’ health, policy, and programming implications**

**Running title:** Victims of war in Uganda

Anastasia Marshak*^a*^*, Teddy Atim*^ab^*, and Dyan Mazurana*^a^*

*^a^* Feinstein International Center, Tufts University, Boston, MA, USA

*^b^* York University, Toronto, Ontario, Canada

*^*^*Corresponding Author:

Tufts University, Feinstein International Center, 75 Kneeland St, 8^th^ floor, Boston, MA, USA.

[anastasia.marshak@tufts.edu](mailto:anastasia.marshak@tufts.edu)

**Data and Methods**

***Timeline***

In Table 1 we provide the timeline of events relevant to the study, including the generation of data used for the analysis.

**Table S1: Timeline of events as it pertains to the ICC trial against Dominic Ongwen**

| *Year* | *Event* |
| --- | --- |
| 1987 | One year after Yoweri Museveni becomes president of Uganda in a civil coup, the voodoo priestess Alice Auma Lakwena takes up arms against the new government. Her movement is defeated at the end of 1987. |
| 1988 | Jospeh Kony, presented as Lakwena’s cousin, takes over the movement which becomes the Lord’s Resistance Army (LRA). Dominic Ongwen is one of the children abducted by the LRA at this time. |
| 1994 | The LRA starts carrying out ambushes and abductions in the north of Uganda. The acts of violence include murder, attempted murder, torture, enslavement, outrages upon personal dignity, pillaging, destruction of property and persecution, forced marriage, rape, sexual slavery, enslavement, forced pregnancy, and conscripting children under the age of 15 [3]. |
| 1999 | Uganda signs the Rome Statute of the ICC. |
| 2001 | The United Nations Human Rights Commission condemns the LRA for forced enrolment of children, kidnappings, torture, detentions, and rapes. |
| 2002 | Uganda ratifies the Rome Statute. |
| 2003 | Dominic Ongwen becomes part of the central command of the LRA. The government of Uganda refers the situation concerning the LRA in northern Uganda to the ICC. |
| 2004 | The LRA attacked Odek, Lukodi, and Abok Internally Displaced Persons (IDP) camps. Ongwen is identified by the ICC as commanding, planning, and coordinating the attacks. The ICC Prosecutor finds grounds to investigate the situation in northern Uganda, which falls within the jurisdiction of the ICC. |
| 2005 | The Office of the Prosecution (OTP) requests arrest warrants for five LRA commanders, including Ongwen. |
| 2007 | The ICC begins to accept victim applications for participation in the case against Ongwen. |
| 2015 | Ongwen is surrendered to ICC custody. Seventy charges were confirmed against Dominic Ongwen by the ICC including attacks against the civilian population in Northern Uganda, murder, attempted murder, torture, cruel treatment, enslavement, pillaging, destruction of property, persecution and other inhumane acts which were allegedly committed in Odek, Abok and Lukodi IDP camps in 2004.  2,605 clients, located in several villages and parishes near Odek, Abok and Lukodi trading centers were granted the right to participate in the case against Ongwen. The clients nominate two lawyers to represent them in the case against Dominic Ongwen referred to as the Legal Representatives for the Victims (LRV). |
| 2016 | The trial against Ongwen begins. |
| 2017 | The LRV hires the services of a team of experts (Dyan Mazurana, Anastasia Marshak, Teddy Atim, and Jordan Farrar) to conduct an independent, in-depth assessment of the victims’ experiences during (2004) and after (2018) the three attacks in Odek, Lukodi, and Abok IDP camps. The Tufts team is selected based on their previous research and academic expertise in northern Uganda. |
| 2018 | February 4^th^ – March 8^th^, the team of experts and authors of this report collect data for the Victimization Survey and lead in partnership with the Secure Livelihoods Research Consortium (SLRC) on data collection in Acholi and Lango sub-region in northern Uganda [38]. The data is immediately analyzed and written up into a report to share with the LRV [36]. |
| 2018 | On May 4^th^, Teddy Atim (a co-author) is called by the LRV as an expert witness at the ICC trial to present the findings of this report. |
| 2021 | On February 4^th^ 2021, the ICC finds Dominic Ongwen guilty of 61 separate war crimes committed in northern Uganda between 1 July 2002 and 31 December 2005 [39]. The Court finds Ongwen guilty, beyond any reasonable doubt, of attacks against the civilian population in the IDPs camps. |

**Data Sources**

In this study we used two data sets, the Secure Livelihoods Research Consortium (SLRC) survey and the Victimization Assessment (VA) Survey to form two study populations: 829 respondents across Acholi and Lango sub-regions representing the general population (GP) and 396 living victim participants (VP).

*Secure Livelihoods Research Consortium (SLRC)*

We collected data on the general population (GP) based on a data collection procedure for the Secure Livelihoods Research Consortium (SLRC) in the Acholi and Lango sub-region in Uganda [32]. Forty surveyors and six team leaders carried out fieldwork between 15 January and 5 March 2018. Preparation for the data collection consisted of a 5-day training. Data collection took 12 days from 20 February to 4 March 2018, using electronic tablets.

To achieve a power of 0.80 while still being representative of the sub-region level (Acholi and Lango), the SLRC study required 40 clusters with at least 768 households per sub-region for a total of 1516 households. We employed a two-stage cluster sample stratified by sub-region strategy to select households and clusters (sub-counties) in the first stage and households within those clusters in the second stage. We used Probability Proportion to Size (PPS) sampling to generate the number of sub-counties sampled in each district for a total of 80 sub-counties or clusters, 40 per sub-district. We selected the sub-counties randomly, and from each sub-county randomly selected one village. In each village, we randomly selected approximately 20 households using a spin the pen approach. The surveyor then requested one household member over the age of 15 to participate in the survey who was able to provide information on behalf of all members of the household.

The qualitative research leads carried out all qualitative interviews. We developed a semi-structured research guide for the interviews and translated the research guide questions from English into the local language, back translated, and pilot tested to ensure accuracy. The surveyors interviewed respondents one-on-one in a private location based on the preference of the respondent.

*Victimization Assessment (VA) Survey*

We carried out the Victimization Assessment (VA) Survey from 20 February to 4 March 2018 with a representative population-based sample of the victim participants (VP) in the case *Prosecutor V. Dominic Ongwen* from Abok, Lukodi and Odek heard by the International Criminal Court. Three camps of Internally Displaced Persons: Abok, Lukodi and Odek were attacked by the LRA under the command of Dominic Ongwen in 2004. The camps are now closed, and the VP population resides in the general community. We selected a subset of the seven best surveyors from the SLRC survey to participate in the VA survey. The preparation for the VA survey data collection consisted of an additional two-day training, with a focus on sensitivity, a new module on psychosocial impact, and some changes to the module on war crimes and crimes against humanity (which we will refer to from now on as simply IHL violations).

The assessment had three objectives: to document the physical, material, and psychosocial harm suffered by the victims as a result of the crimes orchestrated by Ongwen; to assess the immediate and repercussive effects on the victims and members of their households; and to recommend appropriate responses. We used the same wording and questions in the VA survey as in the SLRC survey, with one exception: we amended the wording describing of some of the IHL violations in the VA survey to better capture elements in the ongoing case. In addition, the VA survey added a Psychosocial Assessment. For greater detail regarding the VA Survey please refer to the detailed report [36].

Quantitative sample size for the VA survey required 400 respondents to allow reporting on key outcome indicators with a 5% margin of error and 95% confidence interval. We used a Population Proportional to Size sampling strategy to account for the varying size of client numbers in the three locations. In addition, we stratified the sample by gender to allow disaggregation by gender in the analysis where appropriate. To account for non-response, we selected an additional 5 to 10% of respondents from each site (Table S2). We randomly selected the respondents from the full list of 2605 victims in the case.

We collected the qualitative data through one-on-one, in-depth interviews with 16 of the VP respondents. We selected people from the larger sample who had experienced with different kinds of serious violations of IHL, were of different ages, and included both females and males to ensure a range of different people and experiences. One of the research leads (co-authors) then conducted in-depth interviews. The objectives of the interviews were the same as with the overall study. To reduce possible bias in providing information in the survey, victim participants were not told at any point during the survey that they might be selected for additional interviews.

An important note: the field team had a difficult time reaching 70 of the original respondents randomly selected to take part in the survey. Thus, to reach the desired sample size we randomly selected an additional 70 replacement respondents from the remaining list. While the original selected 70 respondents might have different characteristics than the non-missing sample, the difference would only introduce bias into the data if there was something about these missing respondents that is correlated to more or less experience of serious violations of IHL. However, given the reasons provided by community elders and other community members (sick relatives, schools not in communities, migrated for work, returned to their original homes when they left the IDP camps) as to why some people were not available, we do not find that having experienced more or fewer serious violations of IHL is the primary reason they were unreachable. Thus, we are confident that the final sample of 396 participants was a sound representation of the victim participant community living in this region (Table S2).

The SLRC and VA surveys overlap in timing and geography (Figure S1) allowing for a comparison of the GP and VP. The physical proximity of the VP and GP villages helps support a critical assumption of the analysis that prior to the attacks, the respondents in the VP group were likely similar to the general population.

Figure S1: Map of survey data collection


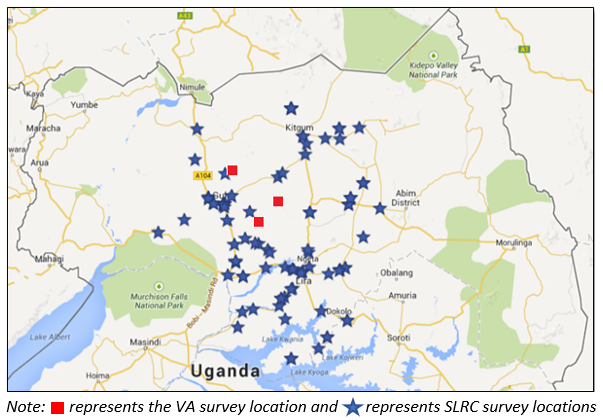


*Ethical procedures*

Across both studies, we obtained informed oral consent from all participants, with opportunities for the interviewee to decline participation prior to and during the interview. We conducted all interviews in private. We removed data identifying the household and kept separate and secure from the data collected on the household. The study team made every effort to ensure confidentiality and to reduce any negative consequences to the participants. Survey participants in the SLRC survey did not receive any material compensation. The study team offered all participants a list and contact information of organizations in their sub-region that specialized in services for victims of violence and made referrals upon request. Tufts University Institutional Review Board (IRB) provided ethical approval for the SLRC study. The Tufts IRB determined that the VA survey did not require IRB approval because data collection took place as part of a court case and not in human subject’s research. However, as we wanted to ensure the safety and rights to voluntary consent of the participants, the team designed and carried out the study in-line with Tufts IRB protocol. Specifically,

- we designed and conducted the study in a manner to minimize risks to research participants;
- ensured equal possibility of participation by all victims with legal status before the Court *in Prosecutor V. Ongwen;*
- informed human subjects of the risks and benefits of participation in the project;
- obtained informed consent from each human research subject prior to beginning the research, documented this;
- confirmed each respondent’s identity through an Legal Representatives for the Victims, but the latter did not attend the interviews nor did the research team have access to the identities of the respondents, just each participant’s unique ID;
- designed and monitored data collection to ensure the safety of the human subjects; and
- made adequate provisions to protect the privacy of human research subjects and to maintain confidentiality of research data.

The research team gave no material incentives to participants other than a bottle of water or soda to drink while they waited, as many had traveled longer distances to reach the sites and no water was available on the sites of the interviews.

*Study outcomes and covariates*

For the comparison of the long-term association of the IHL violations with key outcomes, we focus on the sub-sample of 829 SLRC households (out of 1516) whose members reported *no experience* of IHL violations. The only exception was the experience of the destruction and theft of property. We neither excluded households who experienced this nor counted it (for the purpose of this study only) as a serious violation of IHL because it was experienced by almost all households across the two samples.

All surveys were conducted with the victim respondent. The survey instrument included a roster where we asked the survey respondent to report the following information for themselves but also each of their household members: relationship to household head, age (in years), marital status, highest class of school completed, currently enrolled in school, whether they suffer a physical injury or disability that limits their ability to work and whether that injury was a result of the war. The roster also included questions on experience of serious violations of IHL including: whether a member of the LRA stole their property, a member of the LRA tried to kill them, a member of the LRA inflected several mental or physical pain while they were under their control, a member of the LRA set them on fire, whether they were abducted and whether that abduction occurred when they were underage, whether they were forced to carry out labor when abducted, whether they were forced to kill or seriously injure another person, whether a member of the LRA invaded any opening in their body with any part of their body or an object, whether they were forced to engage in any sexual acts, whether they gave birth to a child from sexual relations with a member of the LRA, whether the LRA deliberately injured their spouse who was not taking part in combat, whether the LRA abducted their spouse, whether the LRA killed their child who was not taking part in combat, whether a member of the LRA abducted their child, whether a member of the LRA killed a close member of their family who was not taking part in combat, whether a member of the LRA deliberately injured a close member of the family who was not taking part in combat, whether a member of the LRA abducted a close member of the family who was not taking part in combat, whether they have been harmed by spirits of the dead.

Disability was assessed using the question in the roster of the respondent and other household members: “do you/does the household member suffer a disability that limits the ability to work?”. We used this question to both assess the proportion of respondents with disabilities and the number of people with disabilities in the household. The dependency ratio was calculated by dividing the number of dependents (children less than 13, adults over 65, and those experiencing disability) by the total household size. Household size was assessed by adding up all members listed in the household roster. Finally, educational attainment was assessed by assigning a number to the highest level of schooling attained.

We assessed ‘access’ to health care facilities using direct questions: “please describe your household’s access to health care services for routine illness or injury” and “please describe your households’ access to health care services for serious illness or injury.”. In both cases we coded the response as “having access” if they responded that they “can access and treatment is available” as opposed to “the treatment we need are usually not available”, “we can access but there is low quality”, “we cannot afford to access healthcare”, “we cannot access healthcare because of distance or transport issues.” Thus, when we use the term ‘access’ in relation to health services we mean a combination of availability, cost, and travel time. We then assessed ‘availability’ using the question: “does the health center or clinic that you attend provide the services and medications your household needs”. We assessed ‘travel time’ with the question “how long does take to reach the health clinic or center that you feel provides adequate care?”. Distance was reported in minutes, irrespective of whether they walked or took transportation.

To assess psychosocial wellbeing, we applied the African Youth Psychosocial Assessment (AYPA) tool [40]. The tool was developed and tested by the Department of Global Health and Population at the Harvard Public Health & Francois-Xavier Bagnoud Center for Health and Human Rights specifically for use among Luo speakers in northern Uganda. The AYPA is comprised of 40 statements that assess a participant’s demeanor over the previous week. Participants are then asked to rate the statement on a scale from 0 to 3, with 0 meaning “Never” and 3 meaning “All the time”. We dropped one question regarding `play with others’ given the age range of the population for a total of 39 statements. The AYPA can be further broken down into four subscales: depression/anxiety (19 statements), conduct problems (10 statements), pro-social behavior (7 statements), and somatic complaints without medical cause (3 statements). The AYPA results in a total AYPA score and a score for each subscale. The total AYPA score includes all subscales except for the prosocial subscale. Therefore, the total AYPA score includes 32 statements and can range from 0 to 96.

To assess the financial capacity to pay for health services, we used the Morris Score Index (MSI), a weighted asset indicator that weighs each durable asset owned by the household by the share of households owning that asset [41]. Assets included: mobile phone, generator, radio, mattress, solar panel, small livestock, medium livestock, large sized livestock, hand tools for digging, hand tools for cutting, plough, powered machinery, bicycle or wheelbarrow, cart for oxen, and motorbike or car. This means that households are considered better off when they owned assets not owned by most households in the sample.

To assess food security, we used the Reduced Coping Strategy Index, based on five coping strategies as proxies for food insecurity [42]. For this study, we calculated the overall score of the insecurity index for each household by multiplying the number of times in the previous week that households used each coping strategy by the pre-assigned weight and summing the products.

To look at long-term effects of the massacre, we looked at current attendance in school of children in the household who were born after the attack on the IDP camps (post 2004). Specifically, we look at whether the child was reported by the respondent to attend school every day. While not perfect, this allows us to look at the association between being born in a household affected by the massacre, but not experiencing the event itself and education outcomes.

We applied population and gender weights to the surveyed VP group to match the distribution by site of the original full list of victims represented in the trial. Specifically, while we designed the sample to include an equal number of men and women, the VA population was 53% female, with slight variation by location. Similarly, we applied weights to each respondent based on each individual’s location so that those from locations with a higher proportion of victims represented by the Legal Representative of the Victims contributed more to the overall means. Given that the sampling strategy for the GP was already representative of the area, we did not assign any weights to the sample.

Table S2: Sample size by survey, site, and gender

| *Survey* | *Site* | *Male* | *Female* | *Total* |
| --- | --- | --- | --- | --- |
| VA Survey | Abok | 67 | 70 | 137 |
|  | Lukodi | 99 | 92 | 191 |
|  | Odek | 33 | 35 | 68 |
|  | Total | 199 | 197 | 396 |
| SLRC | Acholi and Lango sub-region | 296 | 533 | 829 |
| **Total** | | **495** | **730** | **1225** |

*Analysis*

We ran comparison tests using t-tests or z-tests (for continuous and binary outcomes respectively) between all the outcome of interests and whether the respondents was from the VP or GP. Given the importance of sex-disaggregation, comparison tests were also run to compare female GP vs female VP and male GP vs male VP. We also ran four multivariate regressions focused on our health-related outcomes to make sure the significant differences we were seeing in the comparison tests remained once controlling for other factors. To examine the association between the VP and GP and ‘access’ to health services for routine and serious illness as well as whether the appropriate treatment and medicines were available, we applied logistic multivariate regression models for the three outcomes, controlling for sex and age of respondent, their education level, household size, Morris Score Index (i.e. financial capacity), and the Reduced Coping Strategy Index (i.e. food insecurity). For time travelled to health center we used the same regression model but adapted for a continuous outcome. Below is the model specification:

$$Y_{i} = \beta_{0} + \beta_{1}GP+ \beta_{2}[X_{i}]+ \varepsilon_{i}$$

where:

$Y_{i}$ is the outcome for individual or household *i*;

${[X}_{i}]$ is a matrix of control variables for individual or household *i*;

$\varepsilon_{i}$ is the error term for individual or household *i*.

For the binary outcomes, Y takes the logit form, e.g., $\mathrm{logit} \left( P_{i} \right)=\ln\left( \frac{P_{i}}{1-P_{i}} \right)$. All coefficients in the multivariate regression for binary outcomes are reported as odds ratios, meaning the exponentiation of the coefficients of the logit formulation below. To check for possible multicollinearity in the multivariate analysis, we looked at the variance inflation factor and identified no issues of multicollinearity. For all the analysis, we considered a difference with an alpha value of 5% or below to be significant and used STATA 13.

In addition, we ran an analysis using the number of serious violations of IHL as the independent variable on both the Morris Score Index and the AYPA. For the AYPA, we further looked at how each individual serious violation of IHL contributed to the AYPA. We only ran crude models for this analysis using the following model specifications respectively:

$$Y_{i} = \beta_{0} + \beta_{1}(\# of serious violations of IHL )+ \varepsilon_{i}$$

$${AYPA}_{i} = \beta_{0} + \beta_{1}(each indvidual serios violation of IHL)+ \varepsilon_{i}$$

where:

$Y_{i}$ is the YAPA or Morris Score Index for individual or household *i*;

$\varepsilon_{i}$ is the error term for individual or household *i*.
